# Aflibercept monotherapy versus aflibercept with targeted retinal laser to peripheral retinal ischemia for diabetic macular oedema (LADAMO)

**DOI:** 10.1038/s41433-023-02525-9

**Published:** 2023-04-17

**Authors:** E. E. Cornish, S. Wickremasinghe, H. Mehta, L. Lim, S. S. Sandhu, V. Nguyen, M. C. Gillies, S. Fraser-Bell

**Affiliations:** 1 Save Sight Institute, Faculty of Medicine & Health, University of Sydney, Sydney, NSW Australia; 2 Centre for Eye Research Australia, University of Melbourne, Royal Victorian Eye and Ear Hospital, 32 Gisborne Street, East Melbourne, VIC Australia; 3 Ophthalmology Department, Royal Free London, NHS Foundation Trust, London, United Kingdom

**Keywords:** Retinal diseases, Metabolic disorders, Vision disorders

## Abstract

**Objective:**

We tested the hypothesis that targeted retinal laser photocoagulation (TPRP) to peripheral retinal ischaemia reduces the overall burden of aflibercept injections when treating diabetic macular oedema (DMO) over a 24-month period.

**Methods:**

Prospective, double-masked, multicentre, randomised controlled trial in Australia comparing aflibercept monotherapy, following a treat-and-extend protocol, or combination therapy of aflibercept and TPRP for DMO. The aflibercept monotherapy group received placebo laser. The primary outcome measure was the mean number of intravitreal aflibercept injections for each group at 24 months. Secondary outcome included: mean change in central macular thickness (CMT) and vision at trial completion, the proportion of eyes whose DMO resolved and the mean injection treatment interval. Ocular and systemic adverse events were recorded.

**Results:**

We enrolled 48 eyes of 47 patients; 27 eyes were randomised to combination therapy (aflibercept and TPRP) and 21 to aflibercept monotherapy. Thirty-two eyes (67%) completed the 2-year study. The number of intravitreal treatments given were similar for combination therapy (10.5 (SD 5.8) and monotherapy (11.8 (SD5.6)) (*P* = 0.44). The mean visual improvement (+4.0 (−1.8, 9.8) and +7.8 (2.6, 12.9) letters, *P* = 0.32), mean decrease in CMT (−154 (−222,−87) µm and −152 (−218,−86) µm, *P* = 0.96), proportion of eyes with CMT < 300 µm (48% and 67%; P = 0.50) and safety outcomes were similar in both the combination and monotherapy treatment groups (respectively).

**Conclusions:**

Laser to areas of ischaemic peripheral retina does not reduce the burden of intravitreal aflibercept injections when treating diabetic macular oedema.

## Introduction

Diabetic macular oedema (DMO) is the most frequent cause of vision loss in patients with diabetes [1]. It is driven by the release of vascular endothelial growth factor (VEGF) and other inflammatory proteins and cytokines in response to inflammation and retinal hypoxia [2]. VEGF levels are elevated in both the aqueous and vitreous of eyes with DMO [3, 4], suggesting that it is a cause of the increased vascular permeability that leads to DMO. Treatment of DMO with anti-VEGF intravitreal injections is an effective but burdensome treatment, with an average of 15 injections over the first 2 years [5].

Retinal hypoxia elevates levels of VEGF through activation of hypoxia-inducible factor [3, 6]. Diabetic eyes with peripheral non-perfusion are four times more likely to have DMO than eyes without nonperfusion [7]. Hence, we hypothesise that sectoral wide-field angiography guided targeted panretinal photocoagulation laser (TPRP) may reduce the number of VEGF-inhibitor injections for DMO by reducing the hypoxic drive.

The present study treated DMO with aflibercept following a treat-and-extend (T&E) protocol from enrolment. The DAVE Trial randomised 40 eyes with DMO to either ranibizumab *pro re nata* (PRN) alone or ranibizumab PRN with TPRP [8]. The trial found similar injection burden and visual gains for both groups at 3 years. A UK based group conducted a similar trial with 87 eyes treated over one year [9]. Again, the addition of TPRP did not lower the DMO injection burden. We proposed that using a different VEGF-inhibitor without including loading doses in the treatment regimen and extending by 4 weeks, we may see a difference in the number of injections required to treat DMO.

## Materials and methods

### Aim

The specific aim of this trial was to test the hypothesis that targeted laser therapy to areas of peripheral retinal ischemia reduces the overall number of intravitreal aflibercept injections required to control DMO over a 24-month period.

### Outcome measures

The primary outcome measure was the number of intravitreal aflibercept injections in each of the two treatment arms at 24 months. Secondary outcome measures at 24 months compared with baseline included: the proportion of eyes that have central macular thickness (CMT) < 300 µm, mean change in CMT, mean change in best corrected visual acuity (BCVA) and mean treatment interval.

Safety outcomes measures included: the incidence of rescue macular laser, the incidence of ocular adverse events including severe (>15 letter) loss of vision, new proliferative diabetic retinopathy (PDR), cataract progression and the incidence of non-ocular adverse events.

### Patient enrolment

This study was conducted in accordance with the Declaration of Helsinki and was approved by the Human Research Ethics Committees of Sydney Local Area Health Service, the University of Sydney and the Royal Victorian Eye and Ear Hospital (RVEEH) in Melbourne (Protocol number X14-0157). An independent safety monitoring committee reviewed safety data. Patients were recruited from the Sydney Eye Hospital and the RVEEH clinics from 2016 to 2018. All patients had a diagnosis of diabetes mellitus, were older than 18 years and completed written informed consent. Inclusion criteria included: Eyes with CMT > 300 microns on ocular coherence tomography (OCT), BCVA of 35–79 LogMAR letters and peripheral retinal ischemia affecting an area greater than 10-disc diameters on wide-field fundus fluorescein angiogram (FFA). Exclusion criteria included loss of vision due or macular oedema due to other causes, vitreomacular traction or significant epiretinal membrane. Study eye exclusion criteria included: treatment within 6-months with intravitreal triamcinolone or 3-months with anti-VEGF, cataract surgery within 3 months, previous panretinal photocoagulation (PRP) laser or vitrectomy, or media opacity that precluded adequate macular photography. Other exclusion criteria of the patient included pregnancy, unwillingness to use adequate contraception, allergy to fluorescein or aflibercept or intercurrent severe medical diseases.

### Treatment allocation

Each study eye was randomised by the unmasked research officer to either the experimental group “combination therapy” with TPRP and aflibercept, or the active control group “aflibercept monotherapy”. In patients with both eyes eligible for the study, the eyes were randomised to have different therapies.

Stratification to the treatments was carried out to include equal proportions of patients with BCVA less than 70 LogMAR letters at entry to the study. This stratification was inferred from outcomes of DRCRnet Protocol T where participants with worse starting vision had larger visual improvements [5].

### Treatment with aflibercept

Intravitreal aflibercept was administered in a designated treatment facility under sterile conditions as per local site practices. The injection eye was anaesthetised with topical drops and subconjunctival 2% lidocaine. Aflibercept (2 mg/0.05 ml) was injected into the vitreous 4 mm posterior to the limbus using a 30 G needle.

The patient was reviewed four weeks later, and treatment continued following a T&E protocol. Treatment intervals were extended by 4 weeks if VA was ≥84 letters (Snellen equivalent 6/6) or CMT was ≤300 microns. If the treatment interval was greater than 4 weeks but VA was <84 letters or OCT macular thickness was >300 microns, an aflibercept injection was given and the next follow-up interval reduced by 4 weeks.

### Treatment with Targeted peripheral retinal photocoagulation (TPRP)

TPRP was applied with a single spot 532 nm laser to areas of peripheral retinal ischemia in the combination therapy group. No burns were placed within 3000 microns of the optic disc or fovea. Using an appropriate contact lens, the burn size was 400 microns at the retina, with a one-burn width spacing.

In the “combination treatment” group, TPRP was applied 1 month after the initial aflibercept injection. A second was arranged at one months after initial laser depending on the patient’s tolerance of the first laser session. Sham TPRP was applied in the “aflibercept monotherapy” treatment group at 1 months after the initial aflibercept treatment. Patients who received sham laser were laser naïve and did not know about the technique. We turned the laser power down to zero. Laser power was set to zero and no burns were applied. The aiming light and sound still occurred at each pedal press.

### Rescue focal laser

Rescue focal laser could be added to treat the DMO at the investigator’s discretion.

### Data collection and masking

Measurement of BCVA was performed with ETDRS charts using standardized procedures. Cataracts were graded using Age-Related Eye Disease Study photographic standards. CMT was measured from the central 1 mm subfield from spectral domain OCT (Cirrus; Zeiss). Wide-field fundus imaging and FFA was performed (Optos®). Patients underwent dilated fundal examination at each visit. Wide-field FFA was performed at baseline and the Exit visit. At each visit, patients were asked about stroke or heart attack since their last visit. If a patient withdrew their consent, an early exit visit was conducted where possible. When the interval between injections was greater than 3 months, an additional safety visit was arranged. Source data were verified by an independent study monitor of all BCVA and OCT data for all patients.

### Statistical analysis

Data were summarised using the mean, standard deviation (SD), median, first and third quartiles (Q1, Q3) and percentages. Baseline characteristics and 24-month outcomes were compared between the two groups using two sample t-tests, Wilcoxon rank sum tests and Chi-square tests for analysis of means, medians and categorical data, respectively. The time until achieving VA ≥ 85 letters was analysed using Kaplan-Meier survival curves. A *p* value of <0.05 was considered statistically significant. We analysed all eyes regardless of follow up using last observation carried forward. Analyses were conducted using R version 4.0.5 with the survival package (V 3.2–10) for Kaplan–Meier survival curves.

### Power calculations

In Protocol T DRCRnet an average of 15 injections were given over 24 months in the aflibercept group [5]. Whilst there were no inclusion criteria for significant peripheral ischemia, the inclusion and exclusion criteria are otherwise like this study. We assumed that eyes in the monotherapy group would require an average of 15 injections over 24 months, and that eyes in the combination therapy group would require 35% fewer injections than those in the monotherapy group, using a standard deviation of 4.4 injections (interquartile range/1.35 from DRCRnet Protocol T) and a two-sided alpha of 0.05 and power of 80%. Power calculations usually assume a normal distribution, however since the number of injections is count data with a limited and truncated range due to the study protocol, we would expect that the variable will be skewed and increased the sample size to account for this. To detect a difference of 35% in the mean number of injections (between the two treatment groups) assuming standard deviation of 4.4 injections and with two-sided alpha level set at 0.05 and 80% power with the truncated range explained above, a sample size of 20 eyes per treatment arm were required. We also allowed for 10% loss to follow-up per year the rate of loss to follow-up in the BEVORDEX trial [10]. Therefore, a sample size of 24 eyes per treatment arm and 48 eyes in total was chosen.

## Results

**Fig. 1:**
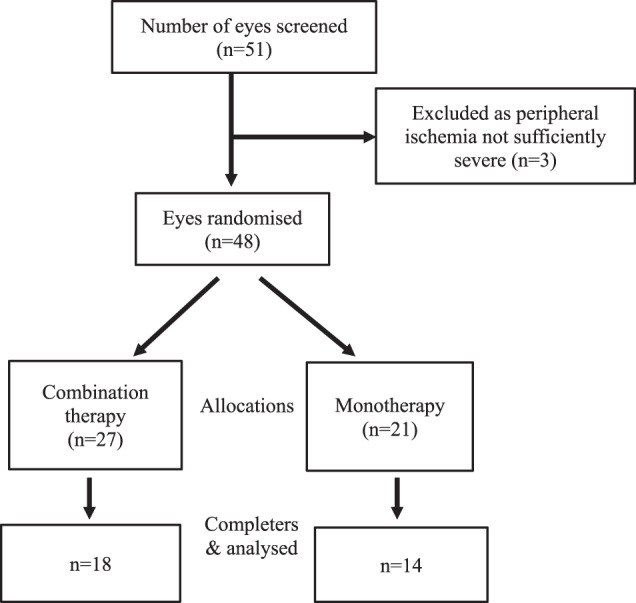
Flow of Patients through the study. Three patients were excluded as their peripheral ischemia was not sufficiently severe enough to reach the inclusion criteria. A total of 48 eyes were included and 32 completed the study.

Fifty-four eyes were screened, 48 eyes were randomised for treatment, 27 to combination therapy and 21 to monotherapy (Fig. 1). Reasons for screen failure were presence of PDR or peripheral retinal ischemia affecting an area less than required for inclusion. Baseline characteristics were similar for each group (Table 1). Sixty-seven percent of the combination therapy eyes and 67% of the monotherapy completed the trial.

**Table 1:** Baseline characteristics.

	Total	Combination therapy	Aflibercept monotherapy	*P* value
Eyes	48	27	21	
Patients	34	26	21	
Age, mean years (SD)	59.9 (10)	60.3 (8.9)	59.3 (11.5)	0.73
Gender, *n* female (%)	16 (33%)	10 (37%)	6 (29%)	0.76
Phakic, *n* (%)	33 (69%)	18 (67%)	15 (71%)	0.97
VA, mean letters (SD)	64.3 (13.1)	63.1 (15)	65.9 (10.2)	0.45
VA, median letters (Q1, Q3)	69 (57, 75)	70 (51, 75)	68 (59, 76)	0.75
≥70 letters, n (%)	24 (50%)	14 (51.9%)	10 (47.6%)	1.00
CMT, mean µm (SD)	483.9 (126.2)	478.9 (136.7)	490 (115.6)	0.77
CMT, median µm (Q1, Q3)	465 (390, 541)	449 (388, 558)	476 (407, 540)	0.57

*CMT* central macular thickness, *Q1* first quartile, *Q3* third quartile, *VA* visual acuity.

### Intravitreal injections

The mean (SD) number of intravitreal injections over the 24 months trial was similar for each group (10.5 (5.8) combination; 11.8 (5.6) monotherapy group (*P* = 0.44)). The mean (SD) treatment interval for each arm was also similar (100.7 days (61.5) combination group and 105.6 days (64.8) monotherapy group (*P* = 0.44)) (Table 2).

**Table 2:** Outcomes of Eyes at 24 months.

	Total	Combination therapy	Aflibercept monotherapy	*P* value
Completers, *n* (%)	32 (67%)	18 (67%)	14 (67%)	1.00
Final VA, mean letters (SD)	70 (16.1)	67.1 (17.8)	73.9 (13.1)	0.13
Final VA, median letters (SD)	71 (62, 83)	70 (56, 81)	77 (66, 84)	0.26
≥70 letters, *n* (%)	27 (56.2%)	15 (55.6%)	12 (57.1%)	1.00
VA change, mean (95% CI)	5.6 (1.8, 9.5)	4 (−1.8, 9.8)	7.8 (2.6, 12.9)	0.32
Gain 5 letters, *n* (%)	28 (58%)	14 (52%)	14 (67%)	0.46
Gain 10 letters, *n* (%)	17 (35%)	9 (33%)	8 (38%)	0.97
Gain 15 letters, *n* (%)	9 (19%)	5 (19%)	4 (19%)	1.00
Final CMT, mean (SD)	317 (99.3)	326.1 (114.7)	306 (78.3)	0.50
Final CMT, median (Q1, Q3)	286 (264, 318)	291 (264, 320)	282 (268, 308)	0.55
<300 microns, *n* (%)	27 (56%)	13 (48%)	14 (67%)	0.45
CMT change, mean (95% CI)	−153.1 (−198.4, −107.8)	−154.1 (−221.6, −86.6)	−151.9 (−218.4, −85.5)	0.96
Injections, mean (SD)	11 (5.7)	10.5 (5.8)	11.8 (5.6)	0.44
Injections, median (Q1, Q3)	10 (7.8, 15)	10 (7, 14)	10 (8, 16)	0.56
Mean injection interval (SD)	102.9 (62.3)	100.7 (61.5)	105.6 (64.8)	0.79
Median injection interval (Q1, Q3)	99.5 (32, 162.8)	91 (42.5, 153.5)	112 (30, 168)	0.87
>15 letter loss of vision	3 (6%)	3 (11%)	0 (0%)	0.25

### Visual acuity

**Fig. 2:**
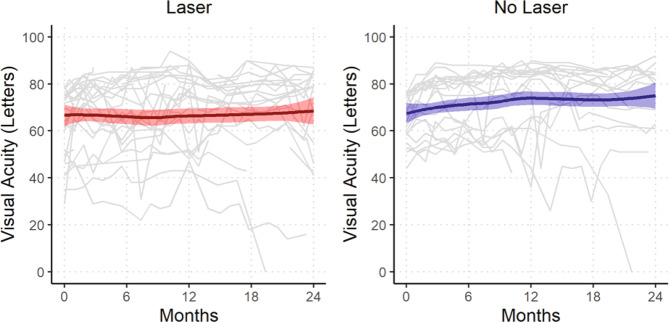
Spaghetti plot showing the visual acuity over 24 months for each eye (grey lines) and the locally weighted scatterplot smoothing (LOESS) curve of the overall trend with the shaded area showing the 95% confidence interval. Analysis was performed on all eyes LOCF.

**Fig. 3:**
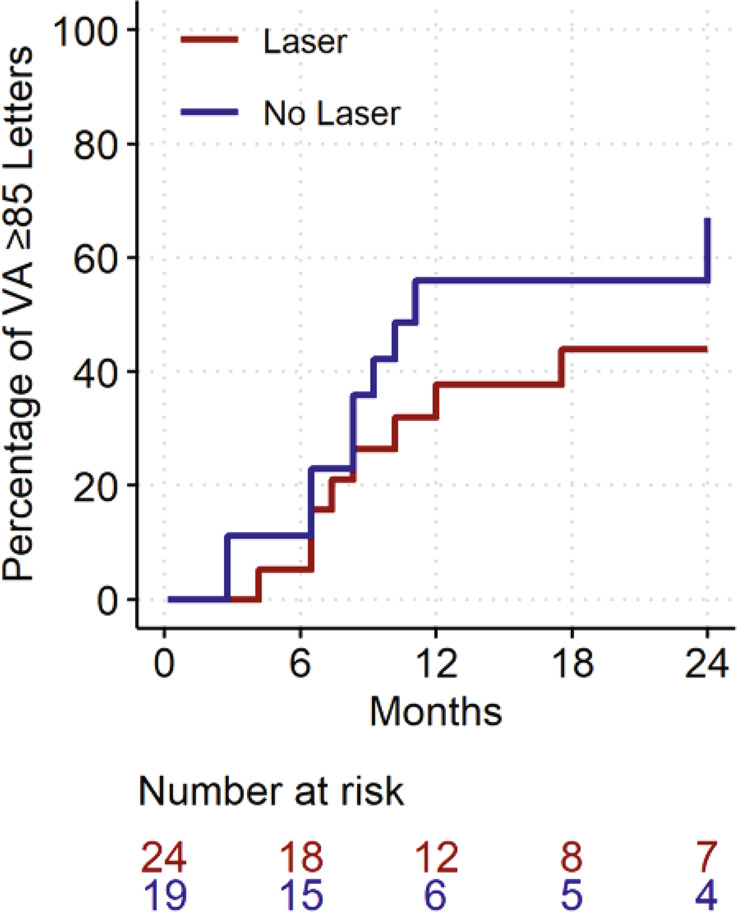
Kaplan-Meier curve of time until achieving VA ≥ 85 letters. Note that it is possible to return below 85 letters by the final visit.

The mean (95% CI) vision change was +4.0 (−1.8, 9.8) letters for combination and +7.8 (2.6, 12.9) letters for monotherapy (*P* = 0.32, Table 2). Fifty-two percent of the combination group and 67% monotherapy group gained at least 5 letters of vision (*P* = 0.46) while 19% of eye in each group gained >15 or more letters (*P* = 1.0). The visual acuity over 24 months for each eye is shown in Fig. 2. Kaplan-Meier curves estimated that that over 40% of eyes in the combination therapy group and over 50% of eyes in the monotherapy group would have vision better than 85 letters BCVA by 2 years (Fig. 3).

### Diabetic macular oedema

Central macular thickness decreased by a mean of 154 µm in the combination therapy and 152 µm in the monotherapy group. Fourteen (67%) and 13 eyes (48%) did not have DMO at end of trial (combination therapy, monotherapy; *P* = 0.45).

### Non-completers

Eleven patients and 16 eyes did not complete the two-year study, 9 eyes from the combined therapy and 7 from the monotherapy arm. The median (Q1, Q3) time to patient drop out was 30 weeks (4, 60) (Suppl. Fig. 1). Non-completers tended to be older, more often male with somewhat worse vison at baseline (Suppl. Table 1). The non-completer eyes had somewhat lower visual gains, higher CMT and received only around half as many injections as the completers (Suppl. Table 2). The numbers of patients that did not complete the study were too few to see significant differences between eyes from each treatment arm.

### Safety outcomes

Rescue focal macular laser was required for two eyes from the combination treatment arm and one eye in the aflibercept only group. One eye from each treatment arm developed a small vitreous haemorrhage due to the injection procedure. No eyes developed endophthalmitis or proliferative diabetic retinopathy. One eye in the monotherapy arm had worsening of cataract. Three eyes (11%) of the combination therapy group had >15 letter loss of vision (*P* = 0.25). The reason for this was cataract progression in one, disease progression (worsening of DMO) in the second and progressive macular ischemia in the third eye.

One patient in the combination therapy group was admitted to hospital for management of ischaemic heart disease. No patient had a cerebrovascular accident.

## Discussion

We found no evidence that the addition of targeted retinal laser photocoagulation to areas of retinal ischemia reduced the treatment burden of DMO compared with intravitreal aflibercept treatment alone. At 24 months, there were no significant differences in the vision gained, nor macular anatomical improvement. The 3-year DAVE trial of 49 eyes, and the 1-year UK based trial of 40 eyes by the RDP study group, found the same outcome as that of our study using ranibizumab instead of aflibercept [8, 9]. We believe that this is due to the high efficacy of VEGF inhibitors on their target in treating DMO as was seen in the 5-year results of Protocol S DRCRnet in which there was significant regression of DR in the actively treated groups [11].

We found no difference in visual gains between either of the treatment arms (+4.0 letters for combination therapy and +7.8 letters for monotherapy (*P* = 0.32). The 1-year UK RDP group trial also found no difference in visual improvement (monotherapy +4 and combination therapy +3.5 letters) [9]. The DAVE trial found final mean visual acuity was similar after 3 years of treatment for each arm of the study (71.6 and 68.2 letters, *P* = 0.45) as we did at 2 years (66.7 and 72.9 letters, *P* = 0.17) (combination therapy, monotherapy) [8]. They theorised that there was no observed benefit of combination treatment because of the higher metabolic demand of the posterior pole, which was not lasered, drives most of the VEGF production rather than the peripheral retinal ischemia [6].

The DAVE trial found greater CMT reduction in the monotherapy group at both 24 (−296 ± 238 µm, −169 ± 172 µm) and 36 months (−302 ± 246 µm, −152 ± 149 µm monotherapy and combination therapy respectively). The authors hypothesised that this was because of heavy laser given at the start and again throughout the trial. In the UK RDP 2000 laser shots were given using the PASCAL laser in one session 2 weeks after the first ranibizumab treatment without further laser treatment. They found that CMT was 316 µm and 311 µm at the end of 1 year of treatment for the monotherapy and combination therapy group (*P* = 0.73). We found that average CMT reduction was similar between monotherapy and combination therapy (−152 µm, −154 µm; *P* = 0.96) and that CMT at end of trial was also not significantly different (306 µm, 326 µm; *P* = 0.55). Similar to the UK RDP group trial, we only performed TPRP at the beginning of the trial.

The mean (SD) number of injections required for DMO in the present study was similar for the monotherapy (11.8 injections) and combination group (10.5 injections) over two years; *P* = 0.44. The UK RDP group trial also found similar number of injections in the two treatment groups over 1 year (6.8 injections, monotherapy; 6.7 injections, combination therapy, *P* = 0.83) [9]. In the RDP trial, three monthly loading doses of ranibizumab was given followed by a PRN regimen. Similarly, the DAVE trial reported that a similar number of injections was given in the monotherapy group compared to the combination therapy group (24.4 injections versus 27.1 injections respectively over 3 years [8].

In summary, we found no effect in TPRP in combination with anti-VEGF therapy in reducing injection load when treating DMO, which is similar to findings from the DAVE and the UK RDP studies, although using a different VEGF inhibitor. Previous 2-year DMO trials have reported a higher number of injections per year than ours, perhaps because our patients were treated with a T&E regimen from the start, whereas other studies begin with a loading course of injections followed by either T&E or pro rata (PRN). DRCR Protocol T used a PRN protocol after a fixed 4 weekly regimen to 6 months and required 15 injections over 2-years (inter quartile range 11–17) [5]. VIVID and VISTA used a fixed interval regimen of 4 or 8 weekly aflibercept injections (after 5 monthly loading doses) and gave 11.8 (2.6) injections (VIVID) and 12.2 (2.6) injections (VISTA) in the first year [12]. The 2-year VIBIM study, which looked at outcomes of DMO using aflibercept and a T&E protocol following five loading treatments 4 weeks apart, reported an average of 12.4 injections over 2 years [13]. Despite fewer mean injections, our study found unequivocal reduction in CMT by 2 years (163 µm in our study compared with 172 µm in the VIBIM study [12], 113 µm in the RETAIN study [13] and 140 µm in the TREX-DMO study [14]) demonstrating a safer treatment paradigm because of fewer treatments when treating with T&E from treatment initiation. We acknowledge that under treatment of DMO is a worldwide issue, however patients in this trial who needed intensive treatment were still able to access it every 4 weeks if required.

A significant weakness of our study is the low study completion rate of 67% (32/48 eyes). Previous DMO studies have had higher rates of retention: DAVE (85%), UK RDP trial (87%), Protocol T (88%), and BEVORDEX (77%). Protocol S had 83% follow-up rates at 2 years and 61% at 5 years [11, 14] A recent urban retrospective study reviewed risk factors of lost to follow up (LTFU) for PDR patients receiving either PRP or IVT [15]. Whilst this was a retrospective chart review rather than a clinical trial it did find a 61% LTFU with an increased risk for patients who had English as the non-primary language (odds ratio 1.83), age over 65 years (OR 1.94), living less than 32 km from the institution (OR 2.68) and having greater than five comorbidities (OR 2.38). Patients that received PRP compared to only intravitreal treatment had a higher rate of LTFU (1.93). Patients from our study were on average 8 years older than those of protocol S (60 vs. 52 years old) and 9 years older than those of CLARITY that had an 86% one-year follow up rate [14, 16]. It is likely our cohort with older aged patients contributed to our poor follow-up rate, in fact the non-completers tended to be older. They also seemed to have more advanced disease as their baseline vision tended to be worse however some may have also been undertreated during the study as they had thicker maculae and more limited visual gains. Although the study was powered, another weakness of the present study is its relatively small patient numbers; we cannot exclude the possibility that a larger, longer study might have found a significant difference between the groups.

Not surprisingly, we found no difference in ocular and systemic safety outcomes for patients in each arm of this study.

Using a treat-and-extend treatment regimen, following an initial loading dose is a commonly used for treatment of neovascular age-related macular degeneration [17, 18]. Clinical trials for diabetic macular oedema trials have typically also included a loading phase of monthly injections, however we question whether this is required for every eye in routine clinical practice. We found in the present study that aflibercept can be safely and effectively used to treat DMO with a T&E protocol from treatment initiation without a loading phase.

In conclusion, we found no reduction in the requirement for aflibercept injections in eyes with DMO and peripheral retinal ischemia when treated with the addition of TPRP. We also found that T&E aflibercept treatment, without a loading phase, is a safe and effective treatment for DMO, leading to fewer clinic visits and a lower treatment burden.

### Summary

#### What was known before

- Targeted peripheral retinal laser (TPRP) photocoagulation with ranibizumab does not reduce the injection burden for patients with diabetic macular oedema compared to monotherapy ranibizumab.
- This was demonstrated with an initial intravitreal treatment with 3 monthly loading phase.

#### What this study adds

- This study tests demonstrated that aflibercept with TPRP to areas of peripheral retinal ischaemia in naive and previously treated eyes does not reduce the injection burden for the treatment of diabetic macular oedema.
- In contrast to the pervious TPRP studies with ranibizumab, this study did not have a loading phase of aflibercept and the treat and extend regimen increased using a 4-week extension interval.
- This increased the potential to find a difference between the number of injections between the two groups but this was not found.

### Supplementary information

Supplementary Figure 1

Supplementary Table 1

Supplementary Table 2

Supplementary Figure Legend

## Data Availability

Data generated during the current study are available from the corresponding author upon reasonable request.

## References

[1] Moss SE, Klein R, Klein BE (1998). The 14-year incidence of visual loss in a diabetic population. Ophthalmology..

[2] Noma H, Yasuda K, Shimura M (2021). Involvement of cytokines in the pathogenesis of diabetic macular edema. Int J Mol Sci.

[3] Aiello LP, Avery RL, Arrigg PG, Keyt BA, Jampel HD, Shah ST (1994). Vascular endothelial growth factor in ocular fluid of patients with diabetic retinopathy and other retinal disorders. N Engl J Med.

[4] Funatsu H, Yamashita H, Sakata K, Noma H, Mimura T, Suzuki M (2005). Vitreous levels of vascular endothelial growth factor and intercellular adhesion molecule 1 are related to diabetic macular edema. Ophthalmology..

[5] Wells JA, Glassman AR, Ayala AR, Jampol LM, Bressler NM, Bressler SB (2016). Afliberc. Ophthalmology..

[6] Arjamaa O, Nikinmaa M (2006). Oxygen-dependent diseases in the retina: role of hypoxia-inducible factors. Exp Eye Res.

[7] Wessel MM, Nair N, Aaker GD, Ehrlich JR, D’Amico DJ, Kiss S (2012). Peripheral retinal ischaemia, as evaluated by ultra-widefield fluorescein angiography, is associated with diabetic macular oedema. Br J Ophthalmol.

[8] Brown DM, Ou WC, Wong TP, Kim RY, Croft DE, Wykoff CC (2018). Targeted retinal photocoagulation for diabetic macular edema with peripheral retinal nonperfusion: three-year randomized DAVE trial. Ophthalmology..

[9] Talks SJ, Bhatia D, Menon G, Cole A, Eleftheriadis H, Downey L (2019). Randomised trial of wide-field guided PRP for diabetic macular oedema treated with ranibizumab. Eye..

[10] Fraser-Bell S, Lim LL, Campain A, Mehta H, Aroney C, Bryant J (2016). Bevacizumab or dexamethasone implants for DME: 2-year results (The BEVORDEX Study). Ophthalmology..

[11] Gross JG, Glassman AR, Liu D, Sun JK, Antoszyk AN, Baker CW (2018). Five-year outcomes of panretinal photocoagulation vs intravitreous ranibizumab for proliferative diabetic retinopathy: a randomized clinical trial. JAMA Ophthalmol.

[12] Korobelnik J-F, Do DV, Schmidt-Erfurth U, David, Holz FG, Heier JS (2014). Intravitreal aflibercept for diabetic macular edema. Ophthalmology..

[13] Kim YC, Shin JP, Pak KY, Kim HW, Sagong M, Lee SJ (2020). Two-year outcomes of the treat-and-extend regimen using aflibercept for treating diabetic macular oedema. Sci Rep.

[14] Gross JG, Glassman AR, Jampol LM, Inusah S, Aiello LP, Antoszyk AN (2015). Panretinal photocoagulation vs intravitreous ranibizumab for proliferative diabetic retinopathy. JAMA..

[15] Green M, Tien T, Ness S (2020). Predictors of lost to follow-up in patients being treated for proliferative diabetic retinopathy. Am J Ophthalmol.

[16] Sivaprasad S, Prevost AT, Vasconcelos JC, Riddell A, Murphy C, Kelly J (2017). Clinical efficacy of intravitreal aflibercept versus panretinal photocoagulation for best corrected visual acuity in patients with proliferative diabetic retinopathy at 52 weeks (CLARITY): a multicentre, single-blinded, randomised, controlled, phase 2b.. Lancet.

[17] Danyliv A, Glanville J, McCool R, Ferreira A, Skelly A, Jacob RP (2017). The clinical effectiveness of ranibizumab treat and extend regimen in namd: systematic review and network meta-analysis. Adv Ther.

[18] Barthelmes D, Nguyen V, Daien V, Campain A, Walton R, Guymer R (2018). Two year outcomes of “treat and extend” intravitreal therapy using aflibercept preferentially for neovascular age-related macular degeneration. Retina..

