# Supplementary figures and images for: Aflibercept monotherapy versus aflibercept with targeted retinal laser to peripheral retinal ischemia for diabetic macular oedema (LADAMO)
Source: Eye (Lond). 2023 Apr 17;37(16):3417–22. doi: 10.1038/s41433-023-02525-9 (PMC10630305; doi:10.1038/s41433-023-02525-9)

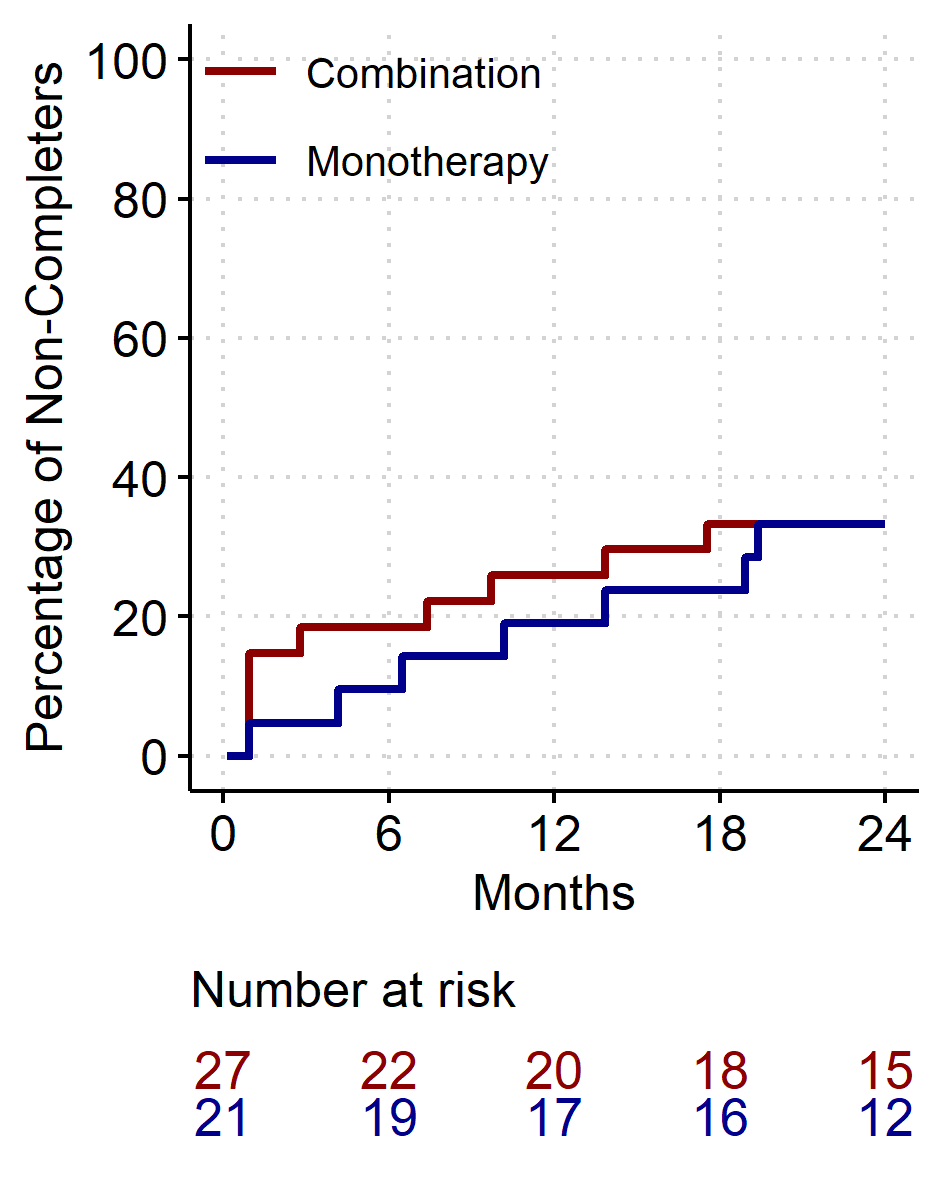

Supplement: Supplementary file 1 — Supplementary Figure 1 [file 41433_2023_2525_MOESM1_ESM.tif]
