# Supplementary material for: Aflibercept monotherapy versus aflibercept with targeted retinal laser to peripheral retinal ischemia for diabetic macular oedema (LADAMO)
Source: Eye (Lond). 2023 Apr 17;37(16):3417–22. doi: 10.1038/s41433-023-02525-9 (PMC10630305; doi:10.1038/s41433-023-02525-9)
Supplement: Supplementary file 2 — Supplementary Table 1 [file 41433_2023_2525_MOESM2_ESM.docx]

|  | **All** | **Combination therapy** | **Monotherapy** | **All non-completers** | **Combination therapy non-completers** | **Monotherapy non-completers** |
| --- | --- | --- | --- | --- | --- | --- |
| **Eyes** | 48 | 27 | 21 | 16 | 9 | 7 |
| **Patients** | 34 | 26 | 21 | 11 | 8 | 7 |
| **Age** | 59.9 (10) | 60.3 (8.9) | 59.3 (11.5) | 65.2 (10.3) | 66.7 (8.6) | 63.4 (12.7) |
| **Female** | 16 (33%) | 10 (37%) | 6 (29%) | 2 (12%) | 1 (11%) | 1 (14%) |
| **Phakic** | 33 (69%) | 18 (67%) | 15 (71%) | 12 (75%) | 6 (67%) | 6 (86%) |
| **VA, letters (SD)** | 64.4 (13.2) | 63.1 (15) | 66.1 (10.5) | 62.8 (12.3) | 61.6 (12.5) | 64.4 (12.8) |
| **VA Median, letters (Q1,Q3)** | 70 (55, 75) | 70 (51, 75) | 70 (58, 76) | 62 (53, 74) | 62 (52, 73) | 70 (56, 75) |
| **VA ≥ 70 letters** | 25 (52.1%) | 14 (51.9%) | 11 (52.4%) | 7 (43.8%) | 3 (33.3%) | 4 (57.1%) |
| **Mean CMT (SD)** | 476 (131.6) | 478.9 (136.7) | 472.4 (128.6) | 478.4 (114.8) | 475.4 (104.5) | 481.8 (136) |
| **CMT Median (Q1, Q3)** | 458 (385, 541) | 449 (388, 558) | 468 (377, 536) | 478 (388, 539) | 478 (396, 522) | 458 (395, 586) |

**Supplementary Table 1**: Baseline characteristics of non-completers of for both Combination therapy and Monotherapy arms.

VA: visual acuity; CMT: central macular thickness; SD: standard deviation;
