# Supplementary material for: Aflibercept monotherapy versus aflibercept with targeted retinal laser to peripheral retinal ischemia for diabetic macular oedema (LADAMO)
Source: Eye (Lond). 2023 Apr 17;37(16):3417–22. doi: 10.1038/s41433-023-02525-9 (PMC10630305; doi:10.1038/s41433-023-02525-9)
Supplement: Supplementary file 3 — Supplementary Table 2 [file 41433_2023_2525_MOESM3_ESM.docx]

**Supplementary Table 2:** Outcomes of non-completers for both combination therapy and monotherapy arms.

VA: visual acuity; CMT: central macular thickness; SD: standard deviation; No.: number

|  | **All** | **Combination Therapy** | **Monotherapy** | **All No-completers** | **Combination Therapy Non-completers** | **Monotherapy Non-completers** |
| --- | --- | --- | --- | --- | --- | --- |
| **Completers** | 32 (100%) | 18 (100%) | 14 (100%) | 0 (0%) | 0 (0%) | 0 (0%) |
| **VA letters (SD)** | 71.6 (17.3) | 68 (20.2) | 76.2 (11.9) | 66.9 (13.3) | 65.2 (12.5) | 69.1 (15) |
| **VA Median (Q1, Q3)** | 80 (64, 84) | 74 (58, 84) | 80 (69, 85) | 70 (60, 78) | 70 (56, 72) | 69 (64, 80) |
| **VA ≥ 70** | 19 (59.4%) | 10 (55.6%) | 9 (64.3%) | 8 (50%) | 5 (55.6%) | 3 (42.9%) |
| **VA Change (Q1, Q3)** | 6.4 (1.1, 11.7) | 4.2 (-4.4, 12.7) | 9.3 (3.1, 15.5) | 4.1 (-1.2, 9.4) | 3.7 (-2.7, 10) | 4.7 (-6.8, 16.2) |
| **VA Gain 15 letters** | 7 (22%) | 4 (22%) | 3 (21%) | 2 (12%) | 1 (11%) | 1 (14%) |
| **CMT (SD)** | 303.5 (99.4) | 311.5 (117.8) | 293.2 (72) | 345.8 (96.1) | 361.6 (106.7) | 330 (89.6) |
| **CMT Median** | 279 (262, 302) | 290 (262, 306) | 278 (264, 283) | 333 (284, 348) | 337 (286, 406) | 331 (283, 336) |
| **CMT 300 µm** | 21 (66%) | 10 (56%) | 11 (79%) | 6 (38%) | 3 (33%) | 3 (43%) |
| **CMT Change** | -151.9 (-200.8, -102.9) | -152.1 (-232.9, -71.2) | -151.6 (-211.7, -91.6) | -156.3 (-276.1, -36.4) | -160.6 (-351.2, 30) | -152.7 (-374.8, 69.4) |
| **No. Injections (SD)** | 13.1 (4.9) | 12.9 (4.7) | 13.3 (5.3) | 7 (5.1) | 5.7 (4.8) | 8.7 (5.2) |
| **No. Injections Median (Q1, Q3)** | 11.5 (9, 16) | 11.5 (9.2, 15.8) | 12 (9, 16) | 6 (2, 10) | 4 (2, 9) | 8 (5.5, 12) |
| **Treatment Interval, days (SD)** | 122.7 (56.9) | 121.6 (54.4) | 124.1 (61.9) | 63.2 (54.5) | 59 (55.3) | 68.6 (57.4) |
| **Treatment Interval Median days (Q1, Q3)** | 121.5 (84, 168) | 113 (84.8, 166) | 131 (91, 168.8) | 32 (28, 83.2) | 32 (28, 56) | 63 (25.5, 83.5) |
| **VA Loss 15 letters** | 3 (9%) | 3 (17%) | 0 (0%) | 0 (0%) | 0 (0%) | 0 (0%) |
