# Supplementary material for: Aflibercept monotherapy versus aflibercept with targeted retinal laser to peripheral retinal ischemia for diabetic macular oedema (LADAMO)
Source: Eye (Lond). 2023 Apr 17;37(16):3417–22. doi: 10.1038/s41433-023-02525-9 (PMC10630305; doi:10.1038/s41433-023-02525-9)
Supplement: Supplementary file 4 — Supplementary Figure Legend [file 41433_2023_2525_MOESM4_ESM.docx]

**Supplementary Figure 1:** Kaplan-Meier curve of time to drop out for non-completers.
